# Molecular identification of CNS NB-FOXR2, CNS EFT-CIC, CNS HGNET-MN1 and CNS HGNET-BCOR pediatric brain tumors using tumor-specific signature genes

**DOI:** 10.1186/s40478-020-00984-9

**Published:** 2020-07-10

**Authors:** Maria Łastowska, Joanna Trubicka, Anna Sobocińska, Bartosz Wojtas, Magdalena Niemira, Anna Szałkowska, Adam Krętowski, Agnieszka Karkucińska-Więckowska, Magdalena Kaleta, Maria Ejmont, Marta Perek-Polnik, Bożenna Dembowska-Bagińska, Wiesława Grajkowska, Ewa Matyja

**Affiliations:** 1 Department of Pathology, The Children’s Memorial Health Institute, Av. Dzieci Polskich 20, 04-730 Warsaw, Poland; 2 Department of Experimental and Clinical Neuropathology, Mossakowski Medical Research Centre, Polish Academy of Sciences, A. Pawińskiego 5 Street, 02-106 Warsaw, Poland; 3 Neurobiology Center, Nencki Institute of Experimental Biology, 2 Pasteur Street, 02-093 Warsaw, Poland; 4 Clinical Research Centre, Medical University of Białystok, Skłodowskiej-Curie 24a Street, 15-276 Białystok, Poland; 5 Clinic of Oncology, The Children’s Memorial Health Institute, Av. Dzieci Polskich 20, 04-730 Warsaw, Poland

**Keywords:** Brain tumors, CNS EFT-CIC, CNS NB-FOXR2, CNS HGNET-MN1, CNS HGNET-BCOR, Genes expression

## Abstract

Four molecular types of rare central nervous system (CNS) tumors have been recently identified by gene methylation profiling: CNS Neuroblastoma with *FOXR2* activation (CNS NB-FOXR2), CNS Ewing Sarcoma Family Tumor with *CIC* alteration (CNS EFT-CIC), CNS high grade neuroepithelial tumor with *MN1* alteration (CNS HGNET-MN1) and CNS high grade neuroepithelial tumor with *BCOR* alteration (CNS HGNET-BCOR). Although they are not represented in 2016 updated WHO classification of CNS tumors, their diagnostic recognition is important because of clinical consequences. We have introduced a diagnostic method based on transcription profiling of tumor specific signature genes from formalin-fixed, paraffin-embedded tumor blocks using NanoString nCounter Technology. Altogether, 14 out of 187 (7.4%) high grade pediatric brain tumors were diagnosed with either of four new CNS categories. Histopathological examination of the tumors confirmed, that they demonstrate a spectrum of morphology mimicking other CNS high grade tumors. However, they also exhibit some suggestive histopathological and immunohistochemical features that allow for a presumptive diagnosis prior to molecular assessment. Clinical characteristics of patients corroborated with the previous findings for CNS EFT-CIC, CNS NB-FOXR2 and CNS HGNET-MN1 patients, with a favorable survival rate for the latter two groups. Among six CNS HGNET-BCOR patients, three patients are long term survivors, suggesting possible heterogeneity within this molecular category of tumors. In summary, we confirmed the effectiveness of NanoString method using a single, multi-gene tumor specific signature and recommend this novel approach for identification of either one of the four newly described CNS tumor entities.

## Introduction

High grade pediatric brain tumors are characterized by a continuing high mortality rate. Some tumors, especially central nervous system primitive neuroectodermal tumors (CNS-PNETs) were challenging to diagnose, being heterogeneous with variable neuronal, ependymal or glial differentiation [3]. Integrated use of technologies for whole genome analysis revealed molecular heterogeneity of CNS-PNETs [16] and the diagnosis of CNS-PNET was removed from the recent 4th revision of WHO Classification of Tumors of the Central Nervous System [9, 11]. Instead, a new diagnostic category of genetically defined embryonal brain tumor was introduced, namely C19MC-altered embryonal tumor with multilayered rosettes (ETMR).

The multicenter study conducted on 323 CNS-PNETs using genes methylation profiling [16] revealed that, on the molecular basis, the majority of CNS-PNETs cluster with other tumors, mainly with high grade gliomas (HGGs), ependymomas (EPNs), medulloblastomas (MBs) or ETMRs. Importantly, four previously unknown types of tumors were discovered, each characterized by the specific recurrent genetic rearrangements:

CNS Neuroblastoma with *FOXR2* activation (CNS NB-FOXR2), CNS Ewing Sarcoma Family Tumor with *CIC* alteration (CNS EFT-CIC), CNS high-grade neuroepithelial tumor with *MN1* alteration (CNS HGNET-MN1), and CNS high-grade neuroepithelial tumor with *BCOR* alteration (CNS HGNET-BCOR). Although the latter four groups were discovered among previously diagnosed CNS-PNETs, subsequent analysis revealed that several tumors were also initially diagnosed as other entities, specifically CNS HGNET-MN1 tumors were classified mainly as EPNs or astroblastomas and CNS HGNET-BCOR tumors as HGGs or MBs [2, 4, 18].

Despite the fact that the newly discovered types of tumors are rare and are not represented in the recent updated edition of WHO 2016 classification, they require diagnostic recognition because of clinical importance. For example, there is a remarkable difference in survival of patients, with poor outcome for CNS HGNET-BCOR tumors and good outcome for CNS HGNET-MN1 and CNS NB-FOXR2 tumors. Following this, de-escalation of treatment for patients with CNS NB-FOXR2 tumors is being considered [6, 8]. Consequently, cIMPACT-NOW (the Consortium to Inform Molecular and Practical Approaches to CNS Tumor Taxonomy) reviewed new CNS tumor types and their nomenclature and recommended that they should be included in the future WHO classification of CNS tumors [10].

Since a diagnosis based on routine histopathology is not feasible, the only reliable and recommended so far method for detection of all four types of tumors was DNA methylation profiling. CNS HGNET-BCOR tumors were also recently diagnosed using capture-based next-generation DNA sequencing [4], nevertheless both methods are still challenging for routine diagnosis.

Importantly, the newly discovered types of tumors also display distinctive gene expression profiles [17]. We have therefore applied an alternative diagnostic method based on transcription profiling of marker genes using NanoString nCounter Technology. This approach allowed for analysis of genes expression from formalin-fixed, paraffin-embedded (FFPE) tumor blocks, routinely prepared in hospitals in everyday practice. We used tumor specific signature genes for identification of all four categories in a series of 187 high grade pediatric brain tumors. We confirmed the effectiveness of this method and performed clinical and histological characteristics of the recently identified types of tumors.

## Materials and methods

### Patients and tumor material

Pediatric patients diagnosed between 1998 and 2019 with high grade brain tumors in The Children’s Memorial Health Institute in Warsaw, Poland, were included in the analysis. The only criterion for inclusion was the availability of tumor material. Only samples examined previously by NanoString method and diagnosed molecularly as MBs [12, 13] were excluded from analysis. Analysis was performed on archive FFPE tumor material obtained at diagnosis and one relapsed sample. Hematoxylin-eosin-stained slides (H&E) were used for pathological evaluation and re-analysis to confirm the original diagnosis and determine the tumor tissue content. Whole preparations were scanned in Hamamatsu NanoZoomer 2.0 RS scanner at the original magnification 40x and analyzed independently by two experienced neuropathologists (W.G. and E.M.). For further investigation only samples with > 70% of tumor cells (90% of samples) and > 60% of tumor cells (10% of samples) were taken into account.

Altogether, 95 cases located in the infratentorial region included 50 EPNs, 37 MBs, 4 choroid plexus carcinomas (CPCs), 3 atypical teratoid rhabdoid tumors (ATRTs) and one glioblastoma (GBM).

Second group of 92 cases located in the supratentorial region included 28 HGGs, 23 PNETs, 20 EPNs, 10 CPCs, 7 ATRTs, 2 ETMRs and 2 tumors classified as CNS embryonal tumors “not otherwise specified” (NOS). Five PNETs were previously analyzed by DNA methylation profiling and presented in the already published series [17]. They included one CNS EFT-CIC, one CNS HGNET-MN1, one CNS HGNET-BCOR and two CNS NB-FOXR2 tumors, therefore we labelled them as the reference samples.

Informed consent was obtained to use tumor material according to the procedures outlined by The Bioethics Committee at the Children’s Memorial Health Institute’s, Warsaw, Poland.

### Identification of marker genes for molecular classification by microarrays in silico analysis

The CEL files deposited in database Gene Expression Omnibus (GEO) were re-analyzed. We selected only pediatric cases (patients < 18 years old) with high grade brain tumors, all analyzed on the platform Affymetrix Human Genome U133 Plus 2.0.

The following datasets were investigated: GSE73038 [17] which included CNS NB-FOXR2, CNS EFT-CIC, CNS HGNET-MN1, CNS HGNET-BCOR, ATRTs, ETMR, HGGs and MBs; GSE70678 [7] which included ATRTs and GSE64415 [15] which included EPNs. CEL files were uploaded to R environment and subsequently normalized by MAS5 method using “affy” library. Next quantile normalization was done separately on infratentorial and supratentorial tumors. Data were log2 transformed and affy probes with the lowest variance (variance< 0.25) were filtered out. In supervised approach, affy probes were selected by Student t-test. A set of samples with histologic diagnosis or a molecular subtype was compared 100 times to a set of the same size of samples drawn from patient’s samples with different histologic  diagnosis or molecular subtype. A mean p-value and mean fold change from 100 t-tests were used as measures of good marker candidate. Random selection of samples and repeating t-test 100 times was done to assure robustness of marker selection.

### Detection of molecular subtypes of tumors at the RNA level

NanoString nCounter system analysis (NanoString Technologies, Seattle, USA) was applied for identification of four molecular groups (CNS NB-FOXR2, CNS EFT-CIC, CNS HGNET-MN1 and CNS HGNET-BCOR) in a series of altogether 187 tumors. Total RNA was extracted from FFPE tumors using RNeasy kits (Qiagen). RNA integrity was assessed using an Agilent 2100 Bioanalyzer.

For group assignment a custom NanoString CodeSet was applied, which consisted of marker genes and three housekeeping genes (*ACTB*, *GAPDH* and *LDHA*).

The sequences of the probes which were designed to target the regions of the marker genes are presented in Additional File 1.

Hybridization of the probes to tumor RNA samples was performed in the Clinical Research Centre, Medical University of Białystok, Poland, according to NanoString Technologies procedures for hybridization, detection and scanning. Raw counts for each gene underwent technical and biological normalization using nSolver software. Clustering of the samples was performed with Euclidean distance metrics and average settings.

### Area under curve (AUC) analysis of the selected marker probes

Nanostring data was uploaded to R and ROCR library was used. Prediction of classes was based on the expression of their marker probes/genes. Performance of the prediction was visualized by the ROC curves, where True positive rate (TPR) is on the y-axis and False positive rate (FPR) on the x-axis.

### Detection of internal tandem duplication in *BCOR* gene

Genomic DNA was extracted from 6 tumor samples, which showed positive HGNET-BCOR signature, using the QIAamp DNA FFPE Tissue Kit (Qiagen). The duplicated region in exon 15 of *BCOR* was detected by targeted PCR using the following primers, as described by Appay et al., 2017 [1]. BCOR_15F: TCCTCCCGCATATTTCGCTG and BCOR_15R: ACACACTGTACATGGTGGGTCC (35 cycles of 98 °C 10s, 60 °C 30s, 72 °C 120 s). Bidirectional sequencing was performed using a 3500 Genetic Analyser (Applied Biosystems, Foster City, CA, USA). The sequences were determined on both DNA strands from at least two independent PCR products. The analyzed sequence fragments were compared with the *BCOR* cDNA (Gen-Bank RefSeq: NM_001123385.1) sequence using Mutation Surveyor software version 3.30 (Soft Genetics, LLC, State Collage, PA, USA).

### Detection of *MN1* fusion by targeted next-generation sequencing

Targeted cancer panel sequencing – the Ampliseq Childhood Cancer Panel for Illumina was used to detect *MN1* gene fusions in four tumors. Prior to the library preparation, total RNA was extracted from FFPE tumor samples using RNeasy Mini Kit (Qiagen) and quantified with QuantiFluor RNA system (Promega). The percentage of RNA fragments > 200 nucleotides using Agilent 2100 Bioanalyzer was calculated (DV200). About 20 ng of RNA was used for library construction according to the manufacturer’s protocol. Each library was qualified using Agilent 2100 Bioanalyzer, and quantified using the QuantiFluor RNA system. The obtained library was then amplified using universal primers targeting the paired-end adapters. Clusters were generated and sequenced on the MiniSeq instrument (Illumina) using the MiniSeq High Output Kit (2 × 150 cycles). Alignment was performed using the Burrows Wheeler Aligner. The BaseSpace RNA Amplicon workflow (Illumina) was used to determine the fusion presence in *MN1* gene.

### Immunohistochemistry

Immunohistochemical reactions were performed on 4 μm thick FFPE sections using the Ventana BenchMark ULTRA IHC/ISH autostaining system. The following Ventana primary antibodies were applied: for Glial Fibrillary Acidic Protein (GFAP), anti-GFAP [clone EP672Y], Rabbit Monoclonal Antibody; for Ki-67, anti-Ki-67 [clone 30–9], Rabbit Monoclonal Antibody, for Olig2, anti-Olig2 [clone EP112], Rabbit Monoclonal Antibody and for Synaptophysin, anti-Synaptophysin [clone MRQ-40], Rabbit Monoclonal Antibody. Antigen retrieval was performed in CC1 buffer followed by detection with the Ultra View HRP system (Roche/Ventana).

Expression of BCOR was detected using commercially available antibody (sc-514,576; Santa Cruz, Dallas, TX), [clone C10] at a dilution of 1:400. Antigen retrieval was performed using Target Retrieval Solution, High pH, (DAKO, Glostrup, Denmark) for 30 min in 99.5 °C. The specificity of BCOR immunoexpression was tested in classic MB and normal testis. Whole slides were scanned in Hamamatsu NanoZoomer 2.0 RS scanner (Hamamatsu Photonics, Hamamatsu, Japan) at the original magnification 40 × .

## Results

### Identification of marker genes for CNS NB-FOXR2, CNS EFT-CIC, CNS HGNET-MN1 and CNS HGNET-BCOR tumors

In the first step, we prepared the list of the top 100 significant genes for one of the four new CNS categories of tumors from the supplementary Table S12 published by Sturm et al., 2016 [17]. Subsequently, from every list we selected the candidates, which were both significantly up-regulated and showed the highest expression level as compared with the all other analyzed types of CNS high grade tumors.

Since > 10% of the cohort analyzed by Sturm et al., 2016 [17] included adult cases, we performed a supplementary analysis of microarray data from pediatric cases only. They included, in addition to the four new CNS tumor entities, also 41 EPNs (GSE64415), 29 HGGs (GSE73038), 12 ATRTs (GSE73038 and GSE70678) and 6 ETMRs, as described in the Material and Methods section. Only supratentorial tumors were analyzed, since CNS NB-FOXR2 and CNS EFT-CIC were not described in the infratentorial region.

Again, we prepare the list of the top 100 significant genes for each category, which were up-regulated at least 10 fold, as compared to all other types of tumors. Of note, among the top 100 significant genes for CNS NB-FOXR2, more than 30% genes were down-regulated, therefore fewer candidate markers for this group could be identified. Following this, a mean p-value and mean fold change from 200 t-tests were used as a measure of good marker candidates for this group. *ECHS1* and *ETV4* genes were not present among the top 100 significant genes from our analysis, nevertheless, we included them to examine their NanoString performance.

Based on the two lists of genes, we finally selected candidate marker genes for each group as presented in Table 1.

**Table 1:** Marker genes selected for identification of the four new CNS categories of tumors

			This study		Sturm et al., 2016
Group	Probe	Gene	Top position significance	Fold	Top position significance
HGNET-BCOR	219433_at	**BCOR**	Group marker		Group marker
HGNET-BCOR	215264_at	EMX1	1	65.5	122
HGNET-BCOR	230311_s_at	PRDM6	3	13.2	47
HGNET-BCOR	230700_at	RTN4RL1	22	12.2	40
HGNET-BCOR	244467_at	SHISA8	29	29.2	12
HGNET-BCOR	214614_at	MNX1	37	27.9	60
HGNET-BCOR	203365_s_at	MMP15	59	10.1	13
HGNET-MN1	1553842_at	**BEND2**	Group marker		Group marker
HGNET-MN1	223347_at	MUM1	2	16	8
HGNET-MN1	207570_at	SHOX	3	362	7
HGNET-MN1	235548_at	APCDD1L	5	185	18
HGNET-MN1	219140_s_at	RBP4	6	158	51
HGNET-MN1	219729_at	PRRX2	10	38.6	112
HGNET-MN1	227194_at	FAM3B	15	320	13
HGNET-MN1	201135_at	ECHS1	–	–	9
NB-FOXR2	1569669_at	**FOXR2**	Group marker		Group marker
NB-FOXR2	239499_at	DNAH2	45	34.5	7
NB-FOXR2	209842_at	SOX10	74	95.9	23
NB-FOXR2	222095_s_at	FAM163A	93	19.8	24
EFT-CIC	212338_at	MYO1D	11	31.7	8
EFT-CIC	209616_s_at	CES1	17	197	43
EFT-CIC	235849_at	SCARA5	44	167	45
EFT-CIC	215161_at	CAMK1G	62	130	1
EFT-CIC	235238_at	SHC4	80	37.5	6
EFT-CIC	1554576_a_at	ETV4	**–**	–	91

### NanoString probes hybridization performance

Marker genes were analyzed separately in supra- and infratentorial locations since the distribution (e.g. MBs, CNS NB-FOXR2 and CNS EFT-CIC) or molecular characteristics (e.g. EPNs) of pediatric CNS tumors is different in these compartments. Therefore, the marker genes for CNS HGNET-BCOR and CNS HGNET-MN1 tumors were analyzed in both supra- and infratentorial location, as opposed to CNS NB-FOXR2 and CNS EFT-CIC tumors, which were described in the supratentorial region only.

Good hybridization was considered when level of expression for the probe in the reference sample was higher (as expected) than mean level of all remaining samples. It ranged between 3.9 fold (for MYOD1) and 92.7 fold (for FOXR2) in our series.

For CNS HGNET-BCOR detection three probes (for *SHISA8, MNX1* and *MMP15*) showed good hybridization in both compartments and additional two probes (for *PRDM6* and *EMX1*) were chosen for detection of supratentorial tumors only, since these genes were over-expressed in several posterior fossa EPNs or MBs. Instead, probe for *RTN4RL1* gene was added to the custom set for detection of posterior fossa CNS HGNET-BCOR tumors.

For CNS HGNET-MN1 detection all six probes in supratentorial tumors (for *BEND2*, *MUM1*, *SHOX*, *APCDD1L*, *FAM3B* and *ECHS1*) showed good hybridization. Only three probes were used in the posterior fossa tumors (for *BEND2*, *SHOX* and *APCDD1L*) and tested positive. Two additional probes examined in the latter compartment (for *RBP4* and *PRRX2*) showed poor hybridization and these genes were excluded from further analysis.

All probes for detection of supratentorial CNS NB-FOXR2 and CNS EFT-CIC tumors showed the good hybridization result.

### Clustering of tumors according to the expression level of marker genes analyzed by NanoString method

**Fig. 1:**
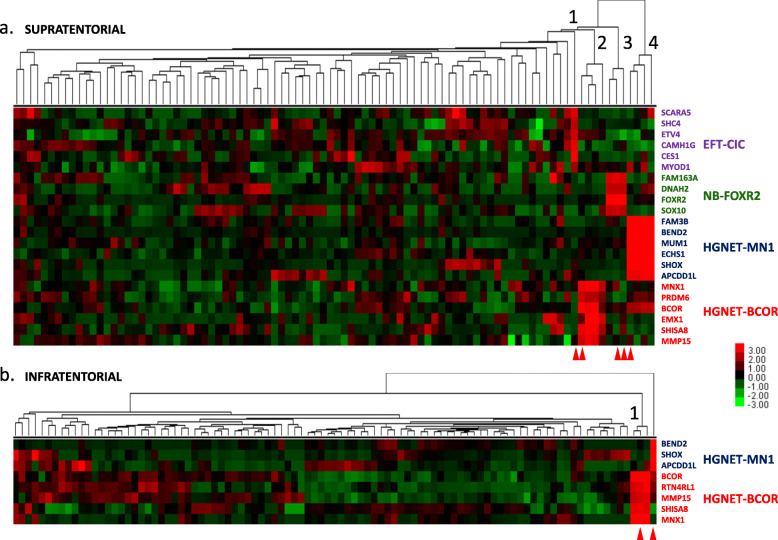
Clustering of brain tumors according to expression level of marker genes using NanoString method. **a** Clustering of 92 supratentorial tumors reveals presence of CNS EFT-CIC (1), CNS HGNET-BCOR (2), CNS NB-FOXR2 (3) and CNS HGNET-MN1 (4) tumors. **b** Clustering of 95 infratentorial tumors reveals presence of CNS HGNET-BCOR tumors (1) and the CNS HGNET-MN1 reference sample. Red arrows indicate location of the reference samples. Colors represent log*2* gene expression differences

Unsupervised hierarchical clustering was performed separately, as mentioned before, for 92 supratentorial and 95 infratentorial tumors (plus two reference samples) with the selected marker genes using Euclidean distance metrics and average settings. The results from supratentorial tumors analysis showed only one reference sample with CNS EFT-CIC signature, three samples with CNS NB-FOXR2 signature (including two reference samples), four samples with CNS HGNET-MN1 signature (including one reference sample) and five samples with CNS HGNET-BCOR signature (including one reference sample), (Fig. 1a). The results from infratentorial tumors analysis showed only one reference sample with CNS HGNET-MN1 signature and three samples with CNS HGNET-BCOR signature (including one reference sample), (Fig. 1b).

### Area under curve (AUC) analysis results for NanoString marker probes

AUC analysis was performed on supratentorial data where all four categories of tumors were detected. Prediction of HGNET-BCOR or HGNET-MN1 or NB-FOXR2 or EFT-CIC classes was based on the expression level of their marker probes presented in Additional File 1. AUC was close to 1 for all the tested markers for classes HGNET-BCOR (6 marker probes), HGNET-MN1 (6 marker probes), NB-FOXR2 (4 marker probes) and EFT-CIC (6 marker probes), (Additional File 2). Close to ideal performance for tested markers may be due to small number of samples of these rare tumors. Especially in case of EFT-CIC subtype only one sample was available. These results indicate that selected NanoString markers can distinguish the individual tumor classes compared to all other classes.

### Further characteristics of CNS HGNET-BCOR tumors

In addition to the reference sample diagnosed previously as PNET, four newly detected CNS HGNET-BCOR tumors were originally diagnosed as HGG. Therefore, we performed further analyses to confirm that their molecular profiles differ from the original diagnosis.

**Fig. 2:**
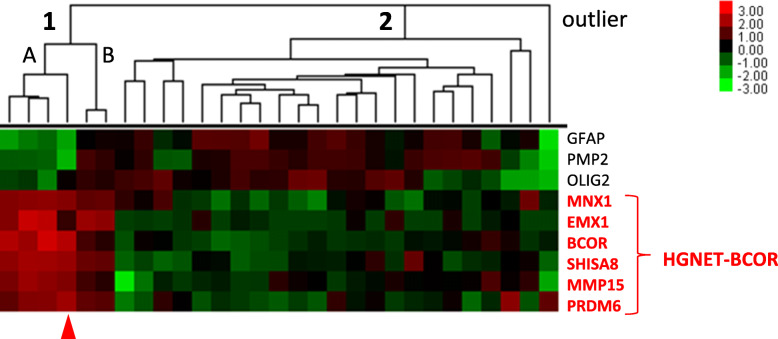
Clustering of tumors with HGG original diagnosis using NanoString method. Cluster 1 represents the samples with high expression of CNS HGNET-BCOR markers. Cluster 2 represents the samples with high expression of glioma markers. Outlier sample is probably not a glioma tumor. Red arrow indicates a location of the CNS HGNET-BCOR reference sample. Colors represent log*2* gene expression differences

We included into the custom set three probes for genes expressed in glioma tumors (*GFAP*, *OLIG2* and *PMP2*), in addition to the CNS HGNET-BCOR marker probes, and performed hybridization in the series of 27 tumors originally diagnosed as HGG and one HGG relapsed sample. NanoString data analysis revealed two distinct clusters and one outlier sample (probably not glioma), (Fig. 2). Cluster 1 included six samples with expressed CNS HGNET-BCOR signature genes and cluster 2 included other samples expressing only *GFAP*, *OLIG2* or *PMP2*. Two samples from the same patient, originally diagnosed as anaplastic oligodendroglioma and the relapsed sample, expressed both CNS HGNET-BCOR signature and glioma marker genes (cluster 1B). Immunohistopathological analysis confirmed positive reaction for expression of both *GFAP* and *OLIG2* in both samples.

Tandem duplication in exon 15 of *BCOR* gene was assessed in all samples diagnosed by NanoString method as CNS HGNET-BCOR. Among 6 tumor samples from cluster 1 (Fig. 2) four cases showed the presence of tandem duplication, including two samples from cluster 1B, one case showed no duplication and in one case the result was inconclusive. Additional CNS HGNET-BCOR cerebellar tumor, originally diagnosed as unclassified malignant neuroepithelial tumor, showed lack of exon 15 of *BCOR* gene duplication and negative reaction for GFAP and synaptophysin.

**Fig. 3:**
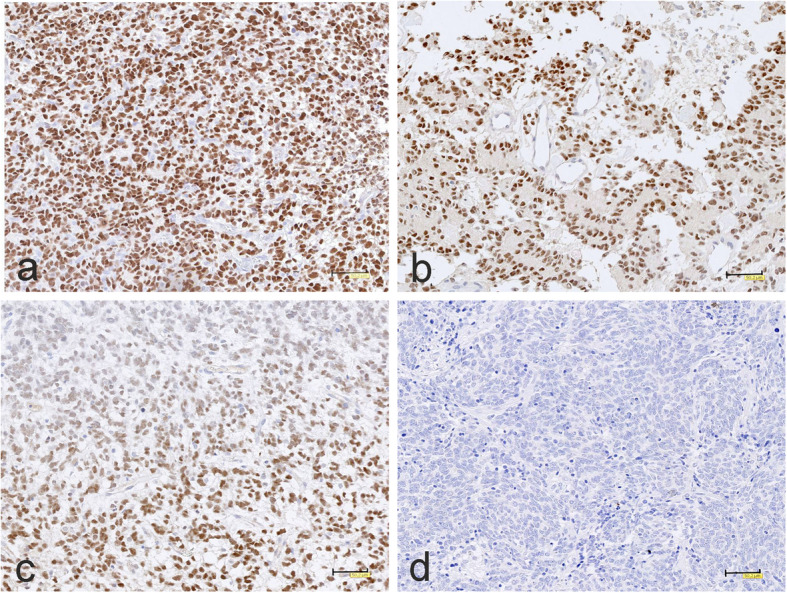
BCOR immunohistochemistry. **a, b** A strong, widespread nuclear BCOR immunereactivity and **c** focal tumor BCOR nuclei staining, in contrast to **d** negative immunostaining of medulloblastoma. The scale bars: a-c – 50 μm

BCOR immunohistochemistry was performed on three tumors with negative or inconclusive *BCOR* tandem duplication results. Two tumors with negative result showed diffuse and strong BCOR nuclear immunopositivity, including tumor with unique papillary architecture originally diagnosed as unclassified malignant neuroepithelial tumor (Fig. 3a,b). The third tumor with inconclusive result revealed only focal BCOR immunopositivity of neoplastic cell nuclei (Fig. 3c). Medulloblastoma tumor, in contrast to CNS HGNET-BCOR tumors, appeared to be BCOR immunonegative (Fig. 3d.)

### Detection of *MN1* fusion transcript

In four tumors showing CNS HGNET-MN1 signature, we were able to perform NGS analysis in order to confirm NanoString results. In all cases the presence of *MN1:BEND2* fusion transcript was detected.

### Histopathological characteristics of tumors

#### CNS EFT-CIC

**Fig. 4:**
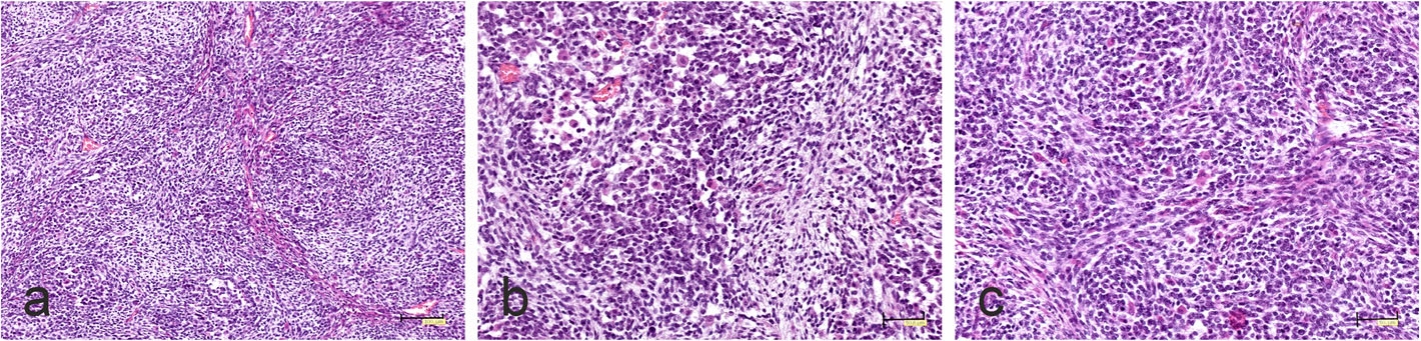
Morphology of CNS EFT-CIC tumor. **a** Densely cellular tumor composed of small, round cells arranged in nodular and fascicular growth pattern. **b** Cells with visible eosinophilic cytoplasm. **c** Focal storiform pattern. The scale bars: a - 100 μm; b, c - 50 μm

Tumor displayed features attributed to originally diagnosed CNS-PNET. It appeared as densely cellular, small cell tumor with nodular, fascicular and focally alveolar growth pattern (Fig. 4a). The nodules were pale and consisted mainly of small round cells exhibiting a neurocytic phenotype. Larger cells with visible eosinophilic cytoplasm were found only sporadically (Fig. 4b). The storiform pattern could be observed focally (Fig. 4c).

#### CNS NB-FOXR2

**Fig. 5:**
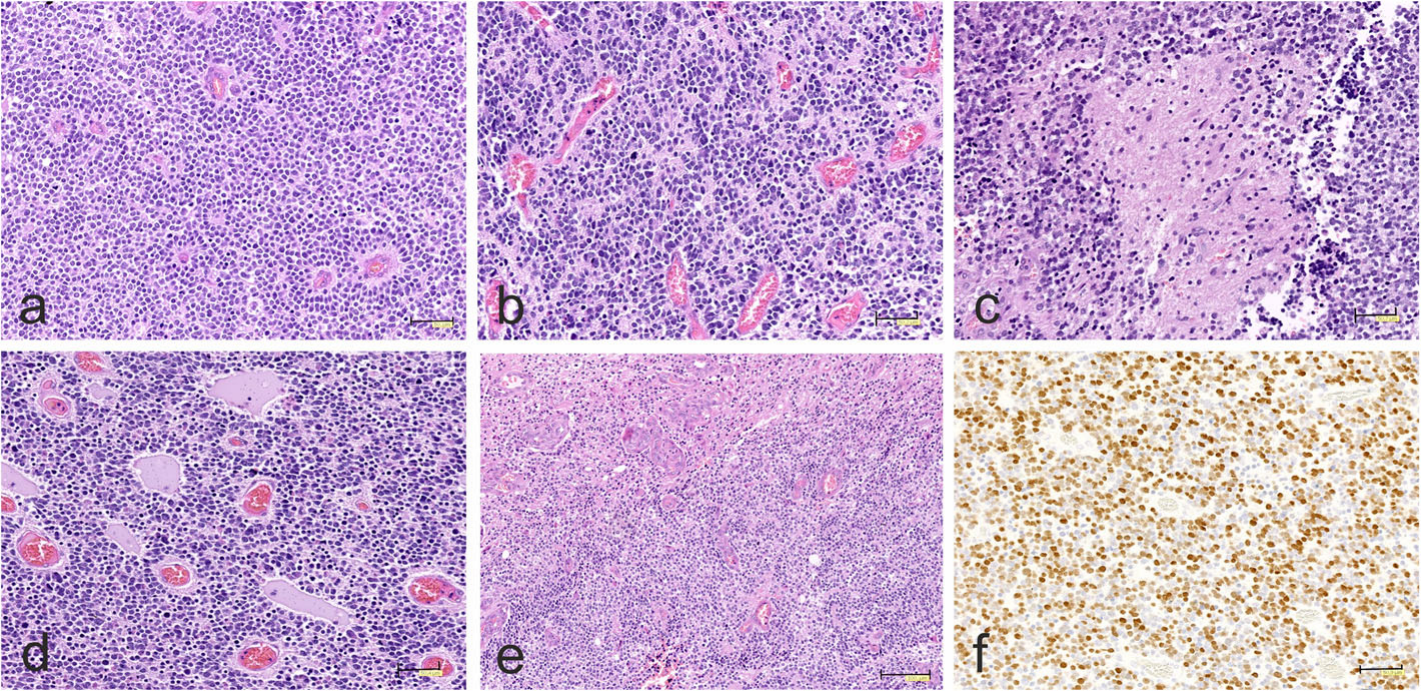
Morphology of CNS NB-FOXR2 tumors. **a** Highly cellular tumor composed of small, round cells with hyperchromatic nuclei surrounded by a clear halo. **b** Densely packed neoplastic tissue with neuroblastic-like rosettes and **c** islands of neuropil. **d** Solid growth pattern with foci of myxoid degeneration. **e** Microvascular proliferation. **f** Strong Olig2 immunoexpression. The scale bars: a-f - 50 μm

All three tumors, originally diagnosed as PNETs, displayed embryonal-like morphology of a highly cellular tumor composed of small, round cells with hyperchromatic nuclei surrounded by a clear halo (Fig. 5a). Occasionally neuroblastic-like rosette formations (Fig. 5b) and neuropil islands (Fig. 5c) could be seen. The tumors were supported by a rich capillary network with presence of pseudorosette arrangement of neoplastic cells. Foci of myxoid degeneration (Fig. 5d) and microvascular proliferation was found (Fig. 5e). The tumor cells exhibited extensive Olig2 immunoexpression (Fig. 5f).

#### CNS HGNET-MN1

**Fig. 6:**
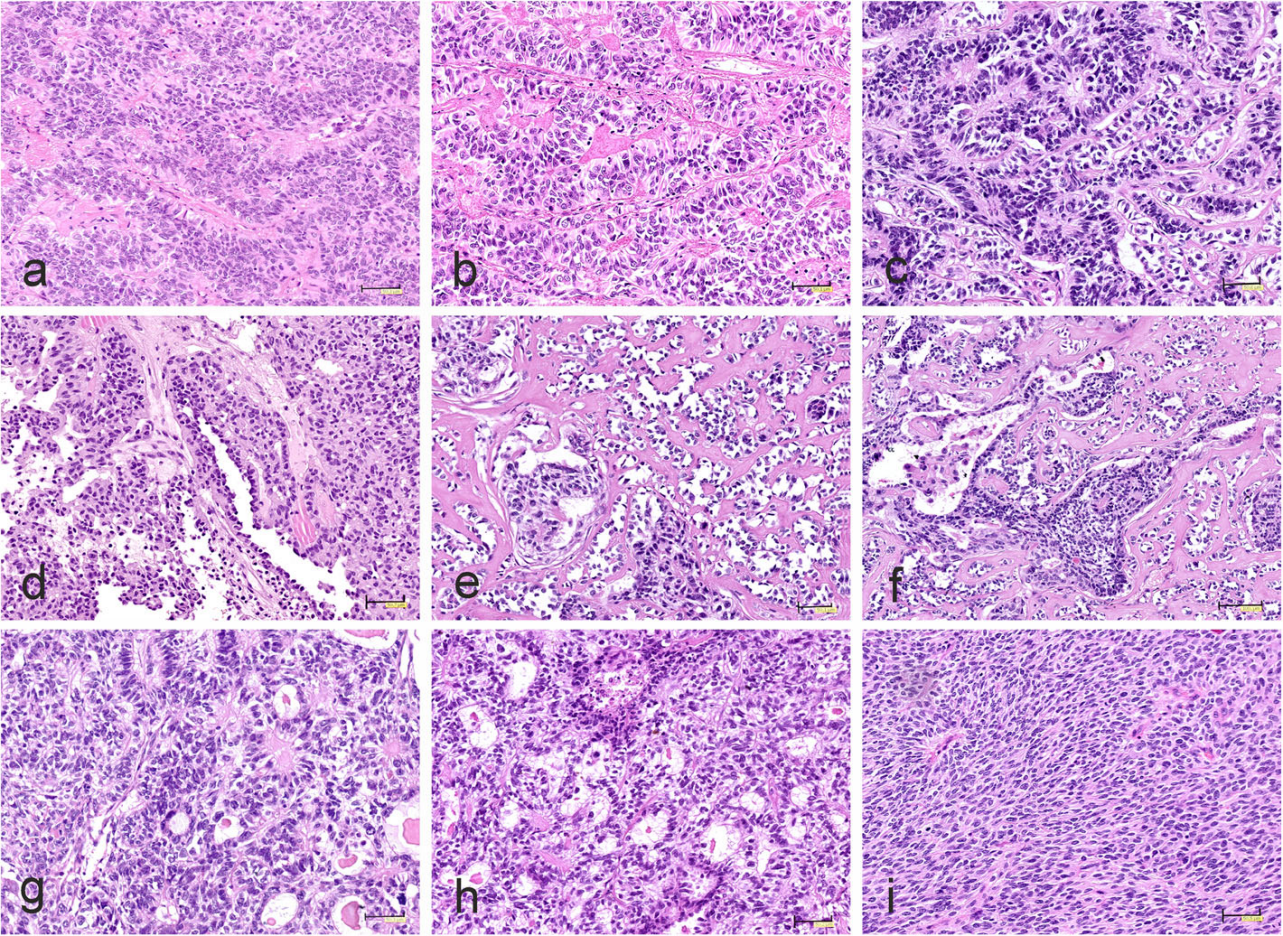
Morphology of CNS HGNET-MN1 tumors. **a** Elongated cells with abundant eosinophilic cytoplasm and prominent processes radiating towards blood vessels. **b** Astroblastoma-type perivascular pseudorosettes with hyaline sclerosis. **c** Perivascular formations **of** ribbon-like appearance. **d** Areas with distinct papillary structure. **e** Advanced hyalinization of vascular stroma and perivascular orientation of neoplastic cells producing a papillary architecture. **f** Extensive hyalinization of fibrovascular stroma. **g, h** The solid growth pattern with papillary formation and microcystic changes. **i** Densely packed, round to ovoid cells with scanty cytoplasm and high mitotic activity. The scale bars: a-e, g-i - 50 μm; f - 100 μm

Four tumors were originally diagnosed as anaplastic EPN (2 cases), CPC (1 case) and PNET (1 case). Most of them exhibited ependymoma-like or astroblastoma-type perivascular pseudorosettes composed of elongated cells with abundant eosinophilic cytoplasm and clear processes radiating towards central blood vessels (Fig. 6a). Pseudorosettes with hyaline sclerosis were commonly observed (Fig. 6b). Perivascular formations formed a focal ribbon-like pattern (Fig. 6c). The tumor initially identified as CPC showed areas with a typical papillary structure (Fig. 6d). A characteristic feature was the extensive hyalinization of the fibro-vascular stroma (Fig. 6e,f). The solid growth pattern with papillary formation (Fig. 6g) and microcystic changes was also seen (Fig. 6h). Only one tumor diagnosed as PNET consisted of densely packed, round to ovoid cells with scanty cytoplasm and high mitotic activity (Fig. 6i). In this tumor, perivascular pseudorosettes have been observed only sporadically.

#### CNS-HGNT BCOR

**Fig. 7:**
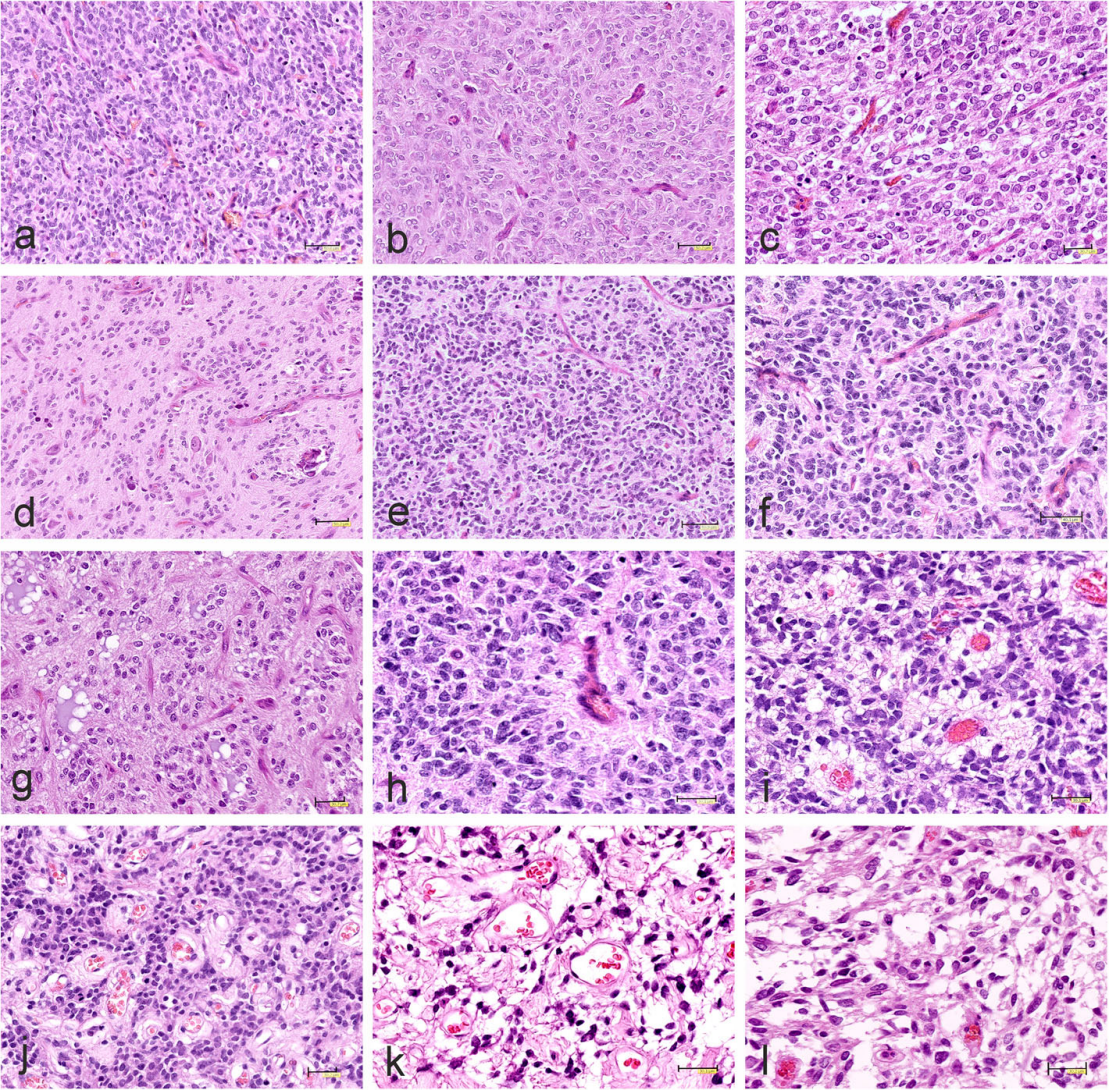
Representative histopathology of CNS-HGNT BCOR tumors. **a** Solid, highly cellular, monomorphic growth pattern with rich, thin-walled capillary network. **b** Pleomorphic cells of astroglial-like appearance. **c** Oligodendroglial-like monomorphic cells with round nuclei surrounded by clear cytoplasm accompanied by chicken-wire pattern of vessels. **d** Infiltration of uniform neoplastic cells with round nuclei and microcalcifications. **e** Densely-packed, small, hyperchromatic cells and thin-walled capillary blood vessels. **f, g** Network of capillary blood vessels with chicken-wire appearance. **h** Perivascular ependymoma-like pseudorosette. **i** Small vessels surrounded by perivascular eosinophilic zones. **j** Hyalinized vessels or **k** thin-walled, round vessels in the spongy matrix. **l** Area composed of stellate cells within myxoid background. The scale bars: a, b, d-e, h, i – 50 μm; f – 40 μm; c, g-l – 30 μm

Three tumors previously identified as GBM exhibited similar morphology with a solid, highly cellular, mostly monomorphic growth pattern (Fig. 7a), accompanied by a rich, thin-walled capillary network.. The majority of neoplastic cells were characterized by ovoid or oval nuclei with fine chromatin and scant eosinophilic cytoplasm with delicate fibrillary processes. Two tumors previously identified as GBM showed an additional pleomorphic cellular component with the appearance of astrocytes or gemistocytes with round to oval nuclei and abundant eosinophilic cytoplasm (Fig. 7b). In one case, the cellular elements contained oligodendroglial-like cells with round nuclei surrounded by clear cytoplasm (Fig. 7c). One tumor diagnosed as pediatric anaplastic oligodendroglioma displayed uniform cells with round nuclei and perinuclear halo, chicken-wire pattern of vessels, numerous persistent neurons with perinuclear satelitosis and microcalcifications (Fig. 7d).

**Fig. 8:**
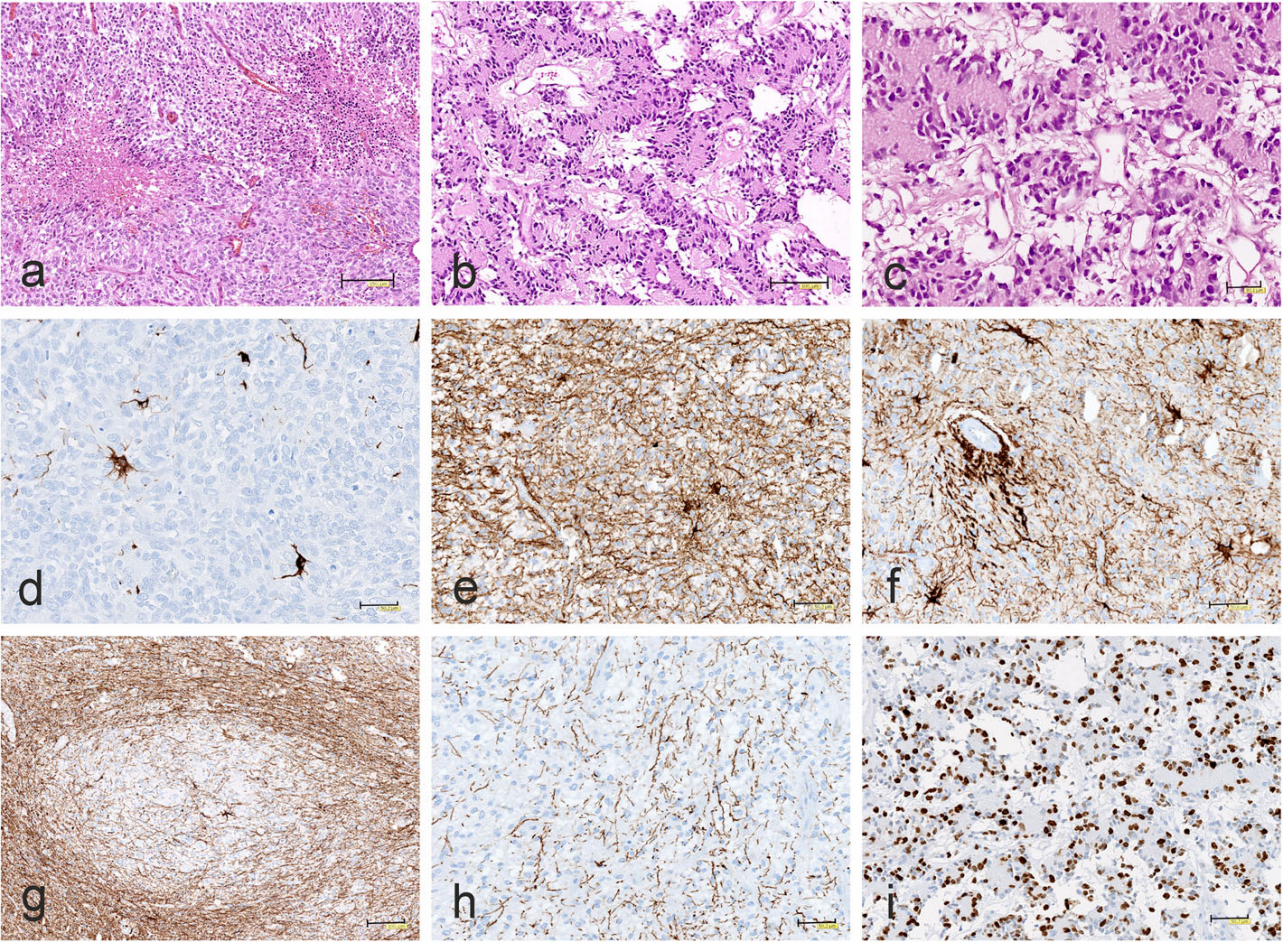
Morphology and immunophenotype of CNS-HGNT BCOR tumors. **a** Micronecrosis. **b** Papillary architecture and microcystic background. **c** Papillary structure with uniform, small cells arranged around eosinophilic acellular cores. **d** A few GFAP-immunopositive reactive astrocytes. **e** Strong GFAP immunoreactivity in reactive astroglial cells and tumor background. **f** Intense GFAP positivity around blood vessels. **g** Focal immunonegativity for GFAP. **h** A few tiny GFAP positive glial processes. **i** High Ki67 proliferation index in unusual malignant papillary neuroepithelial tumor. The scale bars: a - 150 μm; b, g - 100 μm; c - 30 μm; d-f, h, i - 50 μm

Another tumor, initially identified as PNET, showed embryonal-like cytological features with a predominance of densely-packed, small, round, hyperchromatic cells with brisk mitotic activity (Fig. 7e). The most characteristic feature of all BCOR tumors was a distinctive branching network of delicate capillary blood vessels with the dominant chicken-wire appearance (Figs. 7f, g). Perivascular ependymoma-like pseudorosettes were rare (Fig. 7h). Only in the tumor originally considered as PNET, both in the primary tumor and relapse, the small vessels surrounded by perivascular eosinophilic, occasionally microcystic zones (Fig. 7i) and lacking fibrillary processes were more often found. In some areas, the hyalinized vessels (Fig. 7j) or thin-walled, round vessels in the spongy matrix (Fig. 7k) could be observed. The PNET relapse displayed the same morphology as the original tumor. Two cases revealed focal microcystic or cystic changes or areas composed of stellate cells within myxoid background (Fig. 7l). In all cases, micronecrosis (Fig. 8a) and areas of ischemic necrosis were present, sometimes surrounded by pseudopalisading. However, none of the cases identified as HGG showed microvascular proliferation, a key histologic feature of GBM.

The only tumor, initially diagnosed as unclassified malignant neuroepithelial tumor, had a completely different papillary structure (Fig. 8b) with uniform, small cells similar to neurocytes arranged around eosinophilic acellular cores, rarely around blood vessels (Fig. 8c). In some parts of the tumor, the columnar arrangement was seen and the background was partially microcystic. Tumors demonstrated brisk mitotic activity and foci of necrosis.

Immunohistochemically, the majority of CNS HGNET-BCOR tumors did not reveal expression of GFAP, except of some reactive astrocytes (Fig. 8d). Only one tumor, initially diagnosed as pediatric oligodendroglioma, showed strong GFAP immunoreactivity related to numerous entrapped reactive astroglial cells and dense network of their processes in the tumor background (Fig. 8e). Especially intense GFAP positivity was observed around blood vessels (Fig. 8f). However, some areas of tumor were immunonegative for GFAP (Fig. 8g), except a few tiny glial processes (Fig. 8h). In all CNS HGNET-BCOR tumors immunoexpression of synaptophysin was absent and Olig2 immunoreactivity was detected only focally. The Ki67 proliferation index was high in originally diagnosed GBMs and PNET, and also in unusual papillary, neuroepithelial tumor (Fig. 8i).

### Clinical characteristics of patients

In our series, 14 out of 187 (7.4%) patients were diagnosed with either of four new CNS categories of tumors, Table 2. All but two tumors were detected in the supratentorial region.

**Table 2:** Clinical characteristics of patients diagnosed with the four new CNS categories of tumors

ID	Age yrs	Sex	NanoString diagnosis	Orthogonal method	Original diagnosis	Tumor location	Meta-stases	Relapse mts/location	DOD mts	ADF mts	Primary treatment PPNG protocols	
											RT	Chemiotherapy
CIC 1 ref	0.5	f	**EFT-CIC**	Methylation	PNET	fronto-temporal right	no	5/distant	9	–	none	MB/PNET
BCOR 1 ref	1.5	m	**HGNET-BCOR**	Methylation, *BCOR* tandem dup	PNET	frontal right	no	8/local	35	–	none	MB/PNET
BCOR 2	9	m	**HGNET-BCOR**	BCOR immuno	GBM	pons	no	29/local	31	–	local	HGG II version
BCOR 3	6	f	**HGNET-BCOR**	BCOR immuno	GBM	frontal right	no	no	no	144	local	HGG I version
BCOR 4	6	f	**HGNET-BCOR**	*BCOR* tandem dup	GBM	parietal left	no	no	no	168	local	HGG I version
BCOR 5	2.5	f	**HGNET-BCOR**	BCOR immuno	Unclassified malignant neuroepithelial tumor	cerebellar hemisphere	no	no	no	66	CSI	MB SR
BCOR 6	8	m	**HGNET-BCOR**	*BCOR* tandem dup	Anaplastic oligodendroglioma	frontal lobe left	no	22/local	24^tox^	–	local	HGG I version
MN1 ref	2	f	**HGNET-MN1**	Methylation, *MN1:BEND2* fusion	PNET	parieto-temporal right	no	no	no	144	none	MB/PNET
MN2	9	f	**HGNET-MN1**	*MN1:BEND2* fusion	EPN	frontal lobe right	no	no	no	204	local	HGG I version
MN3	11	f	**HGNET-MN1**	*MN1:BEND2* fusion	Papillary EPN	parietal left	no	no	no	132	local	none
MN4	6	f	**HGNET-MN1**	*MN1:BEND2* fusion	CPC	parietal right	no	no	no	180	local	CPC
FOXR2 1 ref	5	f	**NB-FOXR2**	Methylation	PNET	parietal left	yes	no	no	120	CSI	MB/PNET
FOXR2 2 ref	4.5	m	**NB-FOXR2**	Methylation	PNET	parieto-occipital left	no	no	no	137	CSI	MB/PNET
FOXR2 3	7	m	**NB-FOXR2**	nd	PNET	frontal right	no	no	no	240	CSI	MB/PNET

*ref* reference patients, *mts* months, *DOD* Died of disease, *ADF* Alive disease free, *24*^*tox*^ Death from chemotherapy-related toxicity after 24 months, *RT* Radiotherapy, *CSI* Cerebrospinal irradiation, *PPNG* The Polish Pediatric Neurooncology Group, *MB/PNET* Medulloblastoma/PNET protocol, *HGG* High grade glioma protocol,

*MB SR* Medulloblastoma standard risk protocol, *CPC* Choroid plexus carcinoma protocol, *dup* Duplication, *BCOR immuno* BCOR immunohistochemistry, *nd* Not done

Only one patient presented with metastases. Patients were treated according to the Polish Pediatric Neurooncology Group (PPNG) protocols. All patients underwent gross total resection of tumors. The specific protocols for particular patients are listed in Table 2.

Only one infant girl was diagnosed with CNS EFT-CIC (the reference sample). She was treated without radiotherapy during primary treatment, relapsed and died within 1 year from diagnosis.

Three CNS NB-FOXR2 patients (age 4.5, 5, 7 years old) had the previous original diagnosis as PNETs and one patient had metastases to the spinal cord. Because of the same original diagnosis and the similar age, patients were treated according to the same MB/PNET protocol with the cerebrospinal irradiation (CSI). None of the patients relapsed and all are alive at least 10 years since diagnosis.

Four CNS HGNET-MN1 female patients (age 2–11 years old) had different original diagnoses and therefore were treated according to different protocols. However, none of them relapsed and all are long term survivors, with at least 11 years of observation time.

Six CNS HGNET-BCORs patients had various tumor location (four tumors in supra-tentorial region, one in the pons and one in the cerebellar hemisphere). They had also diverse original diagnoses, age (1.5–9 years) and survival rate. Three patients survived at least 5 years without relapse. Notably, two of them, who were treated according to the HGG I version protocol (including Ifosfamide, Etoposide and Adriamycin) are long term survivors, with at least 12 years of observation time.

## Discussion

New molecular categories of tumors discovered by DNA methylation profiling, CNS NB-FOXR2, CNS EFT-CIC, CNS HGNET-MN1 and CNS HGNET-BCOR, are rare pediatric brain malignancies that have not yet been included in the recent WHO 2016 classification. Therefore, reliable identification of new cases diagnosed either retrospectively or prospectively is crucial for a better description of biological and morphological features, as well as clinical characteristics of patients.

In this study, we applied a novel approach for the diagnosis of all four new entities using the NanoString nCounter Analysis System and multi-genes tumor-specific signatures. This method allows for analysis of degraded RNA, is therefore compatible with FFPE specimens and was previously successfully tested for detection of molecular subtypes in MBs [12–14].

We applied a multi-gene detection method with a limited but specific number of probes, what makes such approach both reliable and cost effective. The signatures include group specific marker genes (*BCOR*, *BEND2* and *FOXR2*) which are highly expressed in the relevant groups. All reference RNA samples previously analyzed by DNA methylation profiling clustered within the expected groups, thus validating our NanoString gene expression based diagnosis.

CNS HGNET-MN1 genes (*BEND2, MUM1, SHOX, APCDD1L, FAM3B and ECHS1*) showed robust and clear expression signature. Since no specific immunohistochemical markers were described in this group of tumors, our detection method seems to be of particular diagnostic value. In all four cases analyzed by NGS, the presence of *MN1:BEND2* fusion transcript was detected, again validating NanoString results.

For CNS HGNET-BCOR detection, three probes for *SHISA8*, *MNX1* and *MMP15* showed diagnostic usefulness regardless of tumor location. Multi-gene detection method in this type of tumors can be supplemented by immunohistochemical analysis of BCOR protein. Nevertheless, BCOR can be also expressed in other brain tumors [1, 4, 5], especially in CNS HGNET-MN1, what is visible at the RNA level in our series as well (Fig. 1a).

We should underline, that the described method is based on the open system, where more genes/probes can be added to the custom set. This may be useful for further investigation, especially of CNS EFT-CIC tumors, since only four microarrays profiles were available for identification of marker genes for this group. Also, more genes may be used for better characterization of CNS HGNET-BCOR patients, including potential prognostic markers.

It is important to mention, that the reference samples from the reliable, previously diagnosed tumors should be included in the analysis of new cases. Data from such samples can be stored as RCC files and used for diagnostic comparison. Clustering analysis using freely available nSolver software is rapid and straightforward. In our experience whole diagnostic process from RNA extraction, hybridization and RCC file analysis can be accomplished within 3 days.

We detected altogether 14 samples (7.4%), which showed tumor-specific expression signatures representative for new molecular categories of tumors. Only one CNS EFT-CIC (reference sample) was detected in supratentorial location, confirming very rare occurrence of this type of tumor. This tumor displayed features attributed to originally diagnosed CNS-PNET, as reported in tumors of this molecular group. The cells showed a neurocytic phenotype but lacked immunoexpression of neuronal differentiation.

Three tumors were identified as CNS NB-FOXR2, all originally diagnosed as PNETs, what corroborates with the previous findings [17]. They displayed the same embryonal-like morphology with neuroblastic-like rosettes and pseudorosettes. Foci of myxoid degeneration and microvascular proliferation could be found and the tumor cells exhibited extensive Olig2 immunoexpression. These features correspond to those described so far in CNS NB-FOXR2 tumors. In line with the previous data, all three our patients survived at least 10 years without relapses, what provides further evidence for proposed de-escalation of treatment for this molecular group [6].

Four tumors were identified as CNS HGNET-MN1, all located in the supratentorial region. This suggests that CNS HGNET-MN1 tumors are very rare in the posterior fossa location, in line with the earlier findings, where such location was described in < 10% of CNS HGNET-MN1 tumors by Sturm at al., 2016 [17] and in none out of 8 tumors analyzed by Lehman et al., 2019 [8]. The predominant histologic pattern of HGNET-MN1 tumors, including ependymoma-like or astroblastoma-type perivascular pseudorosettes and hyalinization, is consistent with features previously described in MN1 altered tumors, which were most often identified as astroblastoma. Only one tumor, initially recognized as PNET, consisted of densely packed, small cells with high mitotic activity and inconspicuous perivascular pseudo-rosettes.

Despite different treatment protocols, all four female patients are long term survivors. One patient received local radiotherapy only and another patient chemotherapy only. Since existing data point towards better overall survival rate [6, 9, 17], less intensive therapeutic regiment should be considered for patients with CNS HGNET-MN1 tumors.

Altogether, we have identified 6 CNS HGNET-BCOR cases, four in supratentorial and two in infratentorial location. Three supratentorial tumors harboured *BCOR* exon 15 tandem duplication and in one tumor the result was inconclusive. Two tumors from cluster 1A (Fig. 2), (BCOR2 and BCOR5) lack the presence of duplication despite displaying clear CNS HGNET-BCOR expression signature. An absence of duplication in CNS HGNET-BCOR tumors identified by methylation profiling was described in some cases by Sturm et al., 2016 [17], where an intragenic in-frame deletion in *BCOR* gene or *BCOR* frameshift mutations have been found. Most likely, other rearrangement than *BCOR* exon 15 tandem duplication, are present in BCOR2 and BCOR5 samples.

The majority of our CNS-HGNT BCOR tumors displayed distinctive histological features, including high cellularity, rich capillary network of chicken-wire appearance and palisading necrosis. Most neoplastic cells revealed a glial morphology with eosinophilic cytoplasm and delicate fibrillary processes, less often oligodendroglial-like phenotype with numerous persistent neurons and microcalcifications. The immunophenotype was similar to the previously reported CNS HGNET-BCOR tumors [4]. Majority of currently studied tumors showed only focal positive GFAP staining limited to reactive astrocytes. Even the very strong GFAP expression in the tumor of classic olidendroglial-like histology was associated with the abundant reactive component of stromal astroglia.

Only the case initially diagnosed as unclassified malignant neuroepithelial tumor (BCOR 5) exhibited a completely different morphology, which may indicate some heterogeneity within this molecular group. Because of high Ki67 proliferation index and cerebellar location, this 2.5 year old girl was treated according to the standard risk protocol for medulloblastoma (MB SR) with cerebro-spinal irradiation (CSI), despite young age. She has already survived 5 years without relapse of the disease. By contrast, another young patient (BCOR 1) who did not receive radiotherapy, relapsed within 1 year since diagnosis and subsequently died.

One tumor (BCOR 2) originally diagnosed as GBM was located in the pons and this is only the second CNS HGNET-BCOR described in such location so far.

Two patients also originally diagnosed as GBM (BCOR3 and BCOR4 with confirmed *BCOR* exon 15 tandem duplication) are of particular interest since they did not relapse and are alive at least 12 years since diagnosis. They were treated according to the PPNG HGG I version protocol (including Ifosfamide, Etoposide and Adriamycin). Taking into account the diverse outcome in our patients it seems that CNS HGNET-BCOR group may be clinically and biologically heterogeneous. Indeed, NGS analysis revealed that 40% of analyzed tumors, in addition to *BCOR* exon 15 internal tandem duplication, harbor other likely pathogenic genetic alterations, what may have an impact on tumor behavior [4]. Altogether, CNS HGNET-BCOR category requires further investigation both in terms of detection method and underlying or additional genetic aberrations. It is likely, that methods based on gene methylation or expression profiling detect more CNS HGNET-BCOR tumors than e.g. diagnosis based on detection of *BCOR* exon 15 tandem duplication only.

## Conclusions

We propose a novel approach for identification of either one of four newly described CNS tumor entities using a single multi-gene signature. This method allows also for analysis of FFPE tumor samples routinely preserved in diagnostic laboratories. Our results confirm that the recently proposed molecular classification of malignant pediatric brain tumors demonstrate a spectrum of morphology that mimic other CNS high grade tumors. Nevertheless, they exhibit some suggestive histopathological features and immunohistochemical profile that allow to make a presumptive diagnosis prior to molecular assessment. Further validation and collection of new cases should improve our understanding of the biology of these rare tumors and recommend better therapeutic protocols for patients.

## Supplementary information

**Additional file 1.** The target regions of the marker genes.

**Additional file 2.** Area under curve (AUC) analysis of the marker probes for four category of tumors. Performance of the prediction was visualized by the ROC curves, where True positive rate (TPR) is on the y-axis and False positive rate (FPR) on the x-axis. AUC was close to 1 for all markers tested for HGNET-BCOR (6 marker probes), HGNET-MN1 (6 marker probes), NB-FOXR2 (4 marker probes) and EFT-CIC (6 marker probes) classes.

## Data Availability

All data generated or analysed during this study are included in this published article [and its supplementary information files].

## Abbreviations

ATRTs: Atypical teratoid rhabdoid tumors
CNS: Central nervous system
CNS EFT-CIC: CNS Ewing Sarcoma Family Tumor with *CIC* alteration
CNS HGNET-MN1: CNS high-grade neuroepithelial tumor with *MN1* alteration
CNS HGNET-BCOR: CNS high-grade neuroepithelial tumor with *BCOR* alteration
CNS NB-FOXR2: CNS Neuroblastoma with *FOXR2* activation
CNS-PNETs: Nervous system primitive neuroectodermal tumors
CPCs: Choroid plexus carcinomas
CSI: Cerebrospinal irradiation
EPNs: Ependymomas
ETMR: Embryonal tumor with multilayered rosettes
FFPE: Formalin-fixed, paraffin-embedded
GBM: Glioblastoma
GFAP: Glial Fibrillary Acidic Protein
GSO: Gene Expression Omnibus
H&E: Hematoxylin-eosin-stained slides
HGGs: Grade gliomas
MBs: Medulloblastomas
NOS: Not otherwise specified
