# Supplementary material for: Molecular identification of CNS NB-FOXR2, CNS EFT-CIC, CNS HGNET-MN1 and CNS HGNET-BCOR pediatric brain tumors using tumor-specific signature genes
Source: Acta Neuropathol Commun. 2020 Jul 10;8:105. doi: 10.1186/s40478-020-00984-9 (PMC7350623; doi:10.1186/s40478-020-00984-9)
Supplement: Supplementary file 1 — Additional file 1. The target regions of the marker genes. [file 40478_2020_984_MOESM1_ESM.docx]

Additional file 1. The target regions of the marker genes.

The probes used in Nanostring analysis were designed to target the following regions of the marker genes:

| **Gene** | **Accesson** | **Target sequence** |
| --- | --- | --- |
| **BCOR** | NM_001123383.1 | CACCTCCTCTGTTGGAGAAGCAGACCGTTACCAAAGACGTCACAGATAAGCCACTAGACTTGTCTTCTAAAGTGGTGGATGTAGATGCTTCCAAAGCTGA |
| EMX1 | XM_011532696.1 | GAGGAGGAAGGGCCTGAGTCCGAGCAGAAGAAGAAGGGCTCCCATCACATCAACCGGTGGCGCATTGCCACGAAGCAGGCCAATGGGGAGGACATCGATG |
| PRDM6 | NM_001136239.1 | ATTTAATCAAATCAACGTGAAAAACCAGCGAGTCCTGGCAAGCCCAACTTCCACAAGCCAGCTCCACTCGGAGTTCAGTGACTGGCATCTTTGGAAATGT |
| RTN4RL1 | NM_178568.2 | AGGGGGAGGCAGCAAGAGTCACTTTGGGGGACCAATATTCTTAGATATTTAGAGCATCACCTTGTTTTTATATGCAACACAAGCCTGTCTGCCACCCTGG |
| SHISA8 | NM_001207020.1 | CGCAGCTCCCCGGCCTTTACGGCAGCGCGGGCCGCGGGTCCCGGTACCTAAGGACCAATAGCAAGACCGAGGTCACCGTGTGAAGCGGGGCCGCGGTGCC |
| MNX1 | NM_005515.3 | CCTGGGCGCTTCCCTTTTAAGCAAGGGCGCCTCACCTGCTCTTCAAGAAACAGCGAGAGGGAGACCCAGGGGGCTGAAACTTGAACTCTGGTTCTTTTAA |
| MMP15 | NM_002428.2 | CTGCCCGGTGACATCAGTGCTGCCTACGAGCGCCAAGACGGTCGTTTTGTCTTTTTCAAAGGTGACCGCTACTGGCTCTTTCGAGAAGCGAACCTGGAGC |
| **BEND2** | NM_001184767.1 | GCTCAACTTTATTGGACAGTGACAGTGGCCAGGATTCTTCCTCATCATCTGTCTGTATCCCTCCCAAGTATGGCTATCTTGGTGATCCAAAAAGAAATGT |
| MUM1 | XM_011528382.2 | AAACCCGTGGTAAGTTAAACACACAAATTAGACTGTCTGTGATTTCGAACTTCAGCTGCGGATTTGGGGCTTGGTGAGGAGCAAATACAGATGATTTTGA |
| SHOX | NM_000451.3 | GGAGGGCGGCGGCCACTGCCCGGTGCATTTGTTCAAGGACCACGTAGACAATGACAAGGAGAAACTGAAAGAATTCGGCACCGCGAGAGTGGCAGAAGGG |
| APCDD1L | NM_153360.2 | GCCCAGCAGTCCTGTTCCTCACCCGGCTCTTCACTTTCCACGGGCACAGCCGCTCCTGGGAAGGGTATTACCACCACTTCTCAGACCCAGCCTGCCGGCA |
| FAM3B | NM_206964.1 | GCAGCAAAAGGCTTGGAACTCCCTTCCGAAATTCAGAGAGAAAAGATCAACCACTCTGATGCTAAGAACAACAGATATTCTGGCTGGCCTGCAGAGATCC |
| ECHS1 | NM_004092.3 | GTTCGCTGTCCCGCCTGGCGTCCCTTCGCCTCGGGTGCTAACTTTGAGTACATCATCGCAGAAAAAAGAGGGAAGAATAACACCGTGGGGTTGATCCAAC |
| **FOXR2** | NM_198451.2 | AGGTTTCTCCAGGTCTGACCACTAATCAGTGAGACATTGGTGCCTCTTTCTTCAGTGCTTCTAGCAGGCCTACTGATAAGCTCCCTAGATGAGATACACT |
| DNAH2 | NM_020877.3 | GCCGAATTTGTGTGATGTGGCCCTGGAGATACCTAGTTGTGTTAGCCATAAAAGTGAAAGAGTTGTATTGGAGCTCAGTGCTGTAAAACACCCGCGACAA |
| SOX10 | NM_006941.3 | GAAGCCTCACATCGACTTCGGCAACGTGGACATTGGTGAGATCAGCCACGAGGTAATGTCCAACATGGAGACCTTTGATGTGGCTGAGTTGGACCAGTAC |
| FAM163A | NM_001329714.1 | TATTACTGCTGCAAGAAGAGCGGAACCGAGGTTGCAGACGAGGAGGAGGAGCGGGAGCACGACCTTCCCACGCATCCCAGAGGCCCCACCTGCAATGCCT |
| MYO1D | NM_015194.3 | AAAGTCTCGAGTGATTGTGCAACAGCCAGGAGAAAGAAGCTTTCATTCTTTCTATCAGCTACTCCAAGGAGGTTCAGAACAAATGCTACGCTCTCTACAT |
| CES1 | NM_001266.4 | TCTGTGACCATCTTTGGAGAGTCAGCGGGAGGAGAAAGTGTCTCTGTTCTTGTTTTGTCTCCATTGGCCAAGAACCTCTTCCACCGGGCCATTTCTGAGA |
| SCARA5 | NM_173833.6 | TGGGGCACAGCTCGGGGCCACCCTGACCATGCCTCGACCACACCCCGTCCAGCATTCTCAGTCCTCACACCTGCATCCCAGGACCGTGGGGGCCGGTCGT |
| CAMK1G | NM_020439.3 | CAGGAGCTTTCTCAGAAGTTTTCCTGGTGAAGCAAAGACTGACTGGGAAGCTCTTTGCTCTGAAGTGCATCAAGAAGTCACCTGCCTTCCGGGACAGCAG |
| SHC4 | NM_203349.4 | CAAAGTACACCTGGCTCTGCTGGAAATCAAAGGTCAGCCCAACCACTGGGGAGCCCATGGCACTGCGGAAAGGCACCAGAAACTGTTCAGCCGGGTGCCA |
| ETV4 | NM_001079675.3 | ATGGCTATGAGAAACCTCTGCGACCATTCCCAGATGATGTCTGCGTTGTCCCTGAGAAATTTGAAGGAGACATCAAGCAGGAAGGGGTCGGTGCATTTCG |
