# Supplementary material for: Molecular identification of CNS NB-FOXR2, CNS EFT-CIC, CNS HGNET-MN1 and CNS HGNET-BCOR pediatric brain tumors using tumor-specific signature genes
Source: Acta Neuropathol Commun. 2020 Jul 10;8:105. doi: 10.1186/s40478-020-00984-9 (PMC7350623; doi:10.1186/s40478-020-00984-9)
Supplement: Supplementary file 2 — Additional file 2. Area under curve (AUC) analysis of the marker probes for four category of tumors. Performance of the prediction was visualized by the ROC curves, where True positive rate (TPR) is on the y-axis and False positive rate (FPR) on the x-axis. AUC was close to 1 for all markers tested for HGNET-BCOR (6 marker probes), HGNET-MN1 (6 marker probes), NB-FOXR2 (4 marker probes) and EFT-CIC (6 marker probes) classes. [file 40478_2020_984_MOESM2_ESM.pptx]

## Slide 1
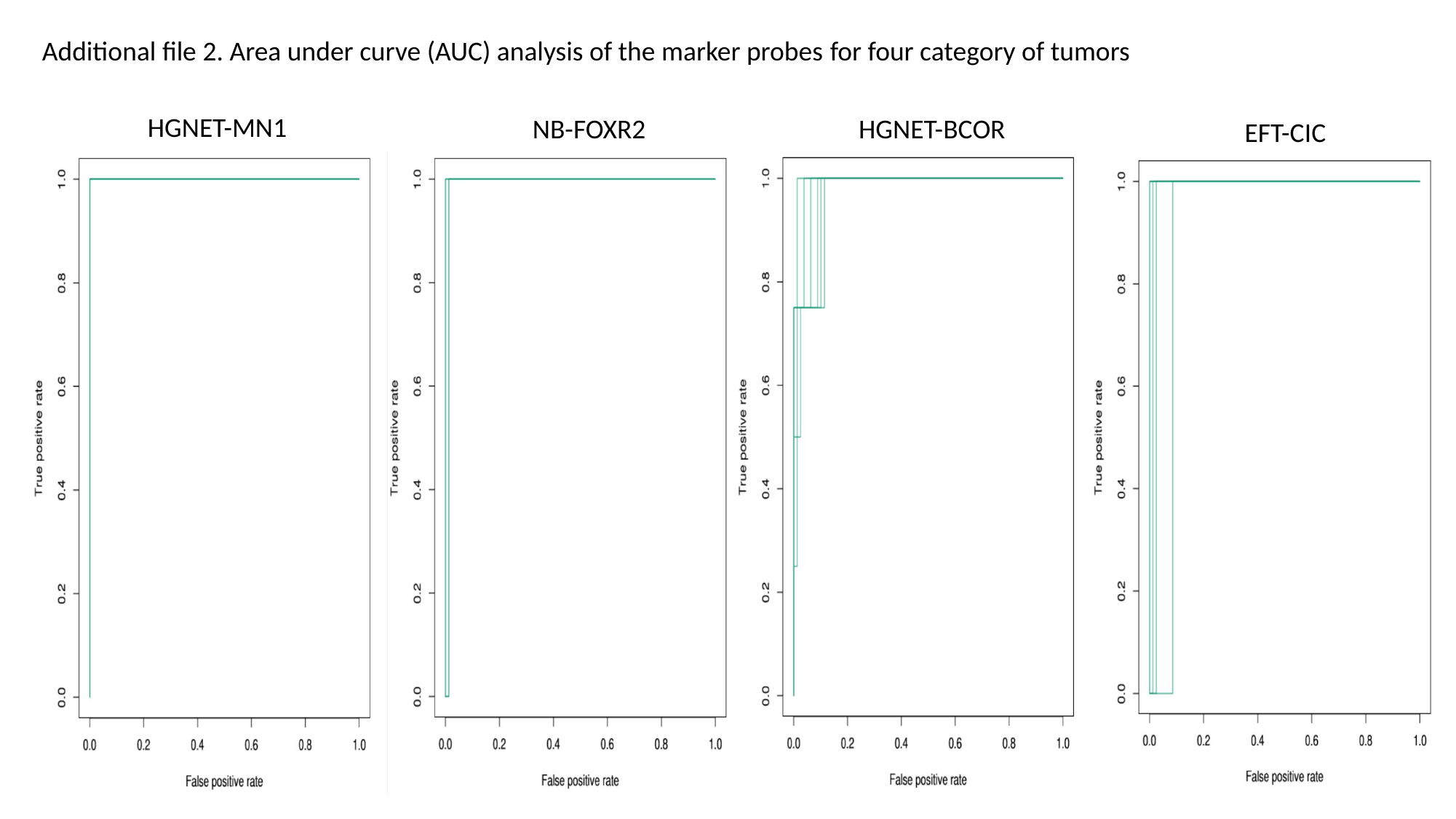

Additional file 2. Area under curve (AUC) analysis of the marker probes for four category of tumors
HGNET-MN1
NB-FOXR2
HGNET-BCOR
EFT-CIC
